# Effect of a 16-Day Altitude Training Camp on 3,000-m Steeplechase Running Energetics and Biomechanics: A Case Study

**DOI:** 10.3389/fspor.2019.00063

**Published:** 2019-11-22

**Authors:** Jean Slawinski, François Chiron, Benjamin Millot, Adrien Taouji, Franck Brocherie

**Affiliations:** ^1^Laboratory Sport, Expertise and Performance (EA 7370), Research Department, French Institute of Sport (INSEP), Paris, France; ^2^Centre de Recherche sur le Sport et le Mouvement - EA 2931, Université de Paris Nanterre, Nanterre, France; ^3^Fédération Française d'Athlétisme, Paris, France

**Keywords:** hurdle, metabolism, kinetics, kinematics, hypoxia, women

## Abstract

The purpose of this study was to investigate the effect of a 16-day training camp at moderate altitude on running energetics and biomechanics in an elite female 3,000-m steeplechase athlete (personal best: 9 min 36.15 s). The 16-day intervention included living and training at 1,600 m altitude. A maximal incremental test was performed at sea level to determine the maximal oxygen uptake ($\overset{∙}{V}O_{2}\max$). Before (pre-) and after (post-) intervention, the participant performed a specific training session consisting of 10 × 400 m with 5 hurdles with oxygen uptake ($\overset{∙}{V}O_{2}$), blood lactate, stride length and stride rate being measured. A video analysis determined take-off distance and landing around the hurdle (DT_H_ and DL_H_), take-off velocity and landing around the hurdle (VT_H_ and VL_H_), and the maximal height over the hurdle (M_H_). The results demonstrated that the mean $\overset{∙}{V}O_{2}$ maintained during the ten 400 m trials represented 84–86% of $\overset{∙}{V}O_{2}\max$ and did not change from pre- to post-intervention (*p* = 0.22). Mean blood lactate measured on the 6 last 400-m efforts increased significantly (12.0 ± 2.2 vs. 17.0 ± 1.6 mmol.l^−1^; *p* < 0.05). On the other hand, post-intervention maximal lactate decreased from 20.1 to 16.0 mmol.l^−1^. Biomechanical analysis revealed that running velocity increased from 5.12 ± 0.16 to 5.49 ± 0.19 m.s^−1^ (*p* < 0.001), concomitantly with stride length (1.63 ± 0.05 vs. 1.73 ± 0.06 m; *p* < 0.001). However, stride rate did not change (3.15 ± 0.03 vs. 3.16 ± 0.02 Hz; *p* = 0.14). While DT_H_ was not significantly different from pre- to post- (1.34 ± 0.08 vs. 1.40 ± 0.07 m; *p* = 0.09), DL_H_ was significantly longer (1.17 ± 0.07 vs. 1.36 ± 0.05 m; *p* < 0.01). VT_H_ and VL_H_ significantly improved after intervention (5.00 ± 0.14 vs. 5.33 ± 0.16 m.s^−^1 and 5.18 ± 0.13 vs. 5.51 ± 0.22 m.s^−1^, respectively; both *p* < 0.01). Finally, M_H_ increased from pre- to post- (52.5 ± 3.8 vs. 54.9 ± 2.1 cm; *p* < 0.05). A 16-day moderate altitude training camp allowed an elite female 3,000-m steeplechase athlete to improve running velocity through a greater glycolytic—but not aerobic—metabolism.

## Introduction

The analysis of the actual male and female 3,000-m steeplechase world records demonstrates that this specific race is ran 3–4% slower than a classical 3,000-m race. This difference in running velocity corresponds to a decrease of ~3% of the oxygen uptake ($\overset{∙}{V}O_{2}$) (Earl et al., 2015). This suggests that 3,000-m steeplechase's pace is near 95% of the athlete's maximal oxygen uptake ($\overset{∙}{V}O_{2}\max$). Thus the ability to elicit a high level of $\overset{∙}{V}O_{2}\max$ during the race is key within the steeplechase performance. However, most of the steeplechase-related studies have focused mainly on the biomechanics of hurdling and water jumps (Hunter and Bushnell, 2006; Hunter et al., 2008; Kipp et al., 2017). Three thousand meter steeplechase athletes compete around the track and have to clear 28 hurdles and seven water jumps over the 3,000-m distance. Therefore, the discipline's performance might depend on technical abilities. Generally, the biomechanical parameters studied are take-off (DT_H_) and landing distances (DL_H_) from the hurdle, time to clear the hurdle (T_H_), take-off velocity (VT_H_) and landing velocity around the hurdle (VL_H_), maximal height over the hurdle (M_H_), stride rate (SR), and stride length (SL), knee and hip angles and/or ground reaction forces (Hunter and Bushnell, 2006; Hunter et al., 2008; Chortiatinos et al., 2010; Hanley and Bissas, 2017; Kipp et al., 2017). A major element to succeed in hurdling events is the athlete's ability to maintain horizontal velocity when they clear the hurdle (Hunter et al., 2008). However, the recent work of Kipp et al. (2017) demonstrated that take-off induces a decrease of the horizontal velocity. This reduction is not compensated after the hurdle, because the horizontal positive impulse increases at landing. In other words, the runner must increase take-off distance in order to clear the barrier as close as possible to the hedge and limit the loss of velocity induced by clearing the hurdle (Hunter et al., 2008). From a kinetic and kinematic point of view, the runner must accelerate before the hurdle in order to increase the vertical forces at take-off and decrease angle at take-off (Salo et al., 1997; Chortiatinos et al., 2010; Kipp et al., 2017). In order to clear a hurdle, a greater muscle activation and force development is needed (Kipp et al., 2017). Therefore, a 3,000-m steeplechase athlete, having 35 hurdles to clear throughout the race needs more force production and probably more anaerobic capacities in comparison to a 3,000-m athlete.

To improve both aerobic and anaerobic capacities in highly-trained endurance runners, living and training at moderate altitude (e.g., 1,600–2,200 m) has been suggested (Gore et al., 2007; Chapman et al., 2014). Recommended procedure to train in altitude were first to decrease the absolute running speed to facilitate the acclimatization process and to have at least 4 weeks of altitude residence (Chapman et al., 2014). Training at $\overset{∙}{V}O_{2}\max$ or anaerobic threshold at moderate altitude (1,400 and 2,100 m) enhances the use of the anaerobic metabolism (Sharma et al., 2019). Indeed, the greater level of muscle deoxygenation induced by hypoxia improved muscle pH regulation, buffer capacity, and anaerobic glycolytic activity (Gore et al., 2007; Sharma et al., 2019). Such adaptations may be particularly effective for 3,000-m steeplechase athletes since both aerobic and anaerobic contributions are required.

The purpose of this study was therefore to test the effectiveness of a 16-day training camp at moderate altitude (1,600 m) on energetics and biomechanics parameters in an elite female 3,000-m steeplechase athlete. We hypothesized that such intervention would improve performance by increasing both aerobic and anaerobic contributions. The increased anaerobic contribution may result in improved hurdling technical ability.

## Materials and Methods

One elite female 3,000-m steeplechase athlete (age: 24 years; height: 172 cm; body mass: 58 kg, personal best: 9 min 36.15 s) gave her informed consent to participate in this study.

### Energetic Parameters

Before intervention, the participant was asked to perform a maximal incremental test (2-min stage) at sea level in order to determine $\overset{∙}{V}O_{2}\max$ and velocity that elicited $\overset{∙}{V}O_{2}\max$ ($\overset{∙}{vV}O_{2}\max$). Before (pre-) and after (post-) intervention, the participant also performed a specific training session consisting of 10 × 400 m with 5 hurdles and a half time effort of passive recovery between each 400-m effort (e.g., a 74 s 400-m run in this case will equal 37 s of passive recovery). This specific training session took place the morning of the day the athlete left sea level and 8 days after her return to sea level. Each 400 m was run at the target velocity of the future 3,000-m steeplechase race pace (5.2 m.s^−1^). During this training session, $\overset{∙}{V}O_{2}$ (in ml.kg^−1^.min^−1^) was continuously measured using a portable unit system (K5, Cosmed Roma, Italy) and calculated as the average $\overset{∙}{V}O_{2}$ of the last 20-s period for each 400 m. This $\;\overset{∙}{V}O_{2}$ was expressed as a percentage of $\overset{∙}{V}O_{2}\max$. Immediately after the 5th, 6th, 7th, 8th, and 9th 400-m effort, blood lactate was measured using a Lactate Pro 2 (Arkray, LT-1730, Kyoto, Japan). For the last 400 m (10th repetition), blood lactate was measured 2 min after exercise cessation.

### Mechanical Parameters

**Figure 1:**
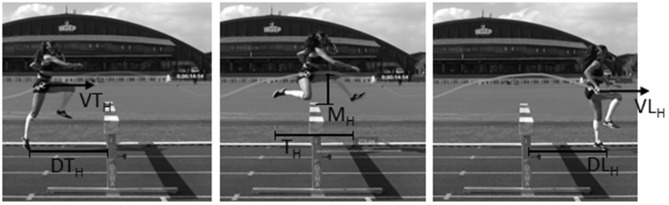
Mechanical parameters recorded during the hurdle clearing. Take-off (DT_H_) and landing distances (DL_H_) around the hurdle, maximal height over the hurdle (M_H_), time to clear the hurdle (T_H_) and take-off (VT_H_) and landing velocities (VL_H_) around the hurdle.

For each 400-m effort, contact time (T_c_), flight time (T_f_), SR, and SL were determined through a 20-m long subsection of the 400 m, using an iPhone SE (240 Hz, Apple, Cupertino, CA, USA) positioned 30 m before the 3rd hurdle. Finally, a fixed video camera (50 Hz, Canon Legria, Paris, France) was placed perpendicularly to the third hurdle in order to determine DT_H_, DL_H_, M_H_, T_H_, VT_H_, and VL_H_ (Figure 1). VT_H_ and VL_H_ were calculated as the average velocity of the step just before take-off and just after landing. All video analysis was realized with Kinovea software (v 0.8.15).

### Moderate Altitude Camp

The participant was then asked to attend a 16-day moderate altitude training camp where she had to live and train at the same altitude (1,600 m). Two daily sessions were implemented throughout the 16-day intervention and were composed of 47% of low-intensity aerobic training (<90% of $v\overset{∙}{V}O_{2}\max$), 9.4% of high-intensity aerobic training (interval training, within 90–110% of $v\overset{∙}{V}O_{2}\max$), 9.4% of very high-intensity lactic training (>110% of $v\overset{∙}{V}O_{2}\max$), 9.4% of resistance training, and 25% of recovery where the athlete had either physiotherapy massage or free time.

### Statistics

In order to compare pre- and post- intervention variables measured during the 10 × 400 m, a parametric student t-test for repeated measures was performed. The level of significance was set at *p* ≤ 0.05.

## Results

### Energetics Parameters

**Figure 2:**
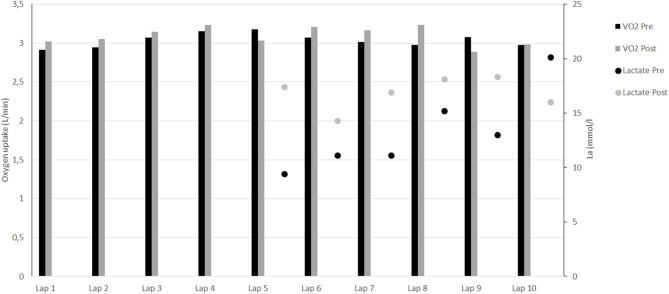
Evolution of oxygen uptake and blood lactate during the 10 × 400-m.

Before intervention, participant's $\overset{∙}{V}O_{2}\max$ and $\overset{∙}{vV}O_{2}\max$ were, respectively 62.1 ml.min^−1^.kg^−1^ and 20.0 km.h^−1^. The mean $\overset{∙}{V}O_{2}$ measured during the ten 400-m efforts did not change from pre- to post- (*p* = 0.22; Figure 2). This represented 84.3 ± 2.4% and 86.0 ± 3.3% of $\overset{∙}{V}O_{2}max\;$for pre- and post-, respectively. The velocity maintained during the 10 × 400 m represented 93.1 ± 0.7% of $\overset{∙}{vV}O_{2}max\;$before intervention and increased significantly to 95.4 ± 1.1% of $\overset{∙}{vV}O_{2}\max$ after intervention (*p* < 0.001). Mean blood lactate measurements from the 5th to the 9th 400-m efforts increased significantly (12.0 ± 2.2 vs. 17.0 ± 1.6 mmol.l^−1^; *p* < 0.05; Figure 2), unlike blood lactate measurements after the 10th 400-m repetition (2 min after exercise cessation) which decreased from 20.1 to 16.0 mmol.l^−1^ following the intervention (Figure 2).

### Mechanical Parameters

Biomechanical analysis revealed that running velocity increased from 5.12 ± 0.16 to 5.49 ± 0.19 m.s^−1^ (*p* ≤ 0.001), concomitantly with SL (1.63 ± 0.05 vs. 1.73 ± 0.06 m; *p* < 0.001). However, SR did not change from pre- to post- (3.15 ± 0.03 vs. 3.16 ± 0.02 Hz; *p* = 0.14). Table 1 presents the evolution of DT_H_, DL_H_ M_H_, T_H_, VL_H_, and VT_H_ from pre- to post-intervention.

**Table 1:** Take-off and landing distances around the hurdle (DT_H_ and DL_H_), maximal height over the hurdle (M_H_), time to clear the hurdle (T_H_) and take-off (VT_H_) and landing velocities (VL_H_) around the hurdle.

	**Pre-**	**Post-**
VT_H_ (m.s^−1^)	5.00 ± 0.14	5.33 ± 0.16^**^
DT_H_ (m)	1.34 ± 0.08	1.41 ± 0.07
M_H_ (cm)	52.5 ± 3.8	54.9 ± 2.1^*^
T_H_ (s)	0.45 ± 0.05	0.46 ± 0.03
VL_H_ (m.s^−1^)	5.18 ± 0.13	5.51 ± 0.22^**^
DL_H_ (m)	1.17 ± 0.07	1.36 ± 0.05^**^

These data were averaged for the 10 laps (^*^p < 0.05; ^**^p < 0.01).

## Discussion

The present study suggests that a 16-day training camp including living and training at moderate altitude (1,600 m) induces a gain in the physiological and biomechanical parameters of a 3,000-m steeplechase specific training session ran by an elite female athlete. Such gains appear to be associated with energetic adaptations—mainly through a higher glycolytic, but not aerobic, metabolism—as well as modifications regarding the biomechanical parameters (i.e., SL, VT_H_, and VL_H_).

The specific training session of 10 × 400 m with hurdles at race pace did not allow the athlete to reach $\overset{∙}{V}O_{2}\max$. Indeed, the average $\overset{∙}{V}O_{2}$ was ~85% of $\overset{∙}{V}O_{2}\max$, although the 400 m was run at 95% of v$\overset{∙}{V}O_{2}\max$. This seems logical since the passive recovery allowed between each 400 m effort prevented the athlete to reach $\overset{∙}{V}O_{2}\max$ during such specific session. The inclusion of hurdles generally results in an increase of the hurdle's approach velocity (Earl et al., 2015) as well as a decrease of the average running velocity (3,000-m steeplechase is 30 s slower compared to a classical 3,000 m; Chortiatinos et al., 2010). Therefore, increase in $\overset{∙}{V}O_{2}$ between 3,000-m steeplechase and classical 3,000-m is small and non-significant (Earl et al., 2015). Thus, the decrease of running velocity during a 3,000 m steeplechase may compensate a part of the increase in the energetic demand requested for hurdling technique. This discipline requires to clear 35 hurdles, therefore, the anaerobic capacity might be more solicited than during a classical 3,000-m race (Kipp et al., 2017). The 16-day training camp at moderate altitude training might have an impact on this anaerobic capacity.

The general consensus about living and training at altitude method is that altitude chronic exposure (residence) and increased relative intensity of training induced by hypoxic training result in physiological and performance improvements (Saunders et al., 2009; Pugliese et al., 2014; Solli et al., 2017). According to the meta-analysis from Bonetti and Hopkins (2009), the velocity maintained during the 10 × 400-m increased by 2.4%, meanwhile the intervention did not induce any change in $\overset{∙}{V}O_{2}$. This result appears in line with previous studies, suggesting that training at moderate altitude during a relatively short period (16 days) does not lead to $\overset{∙}{V}O_{2}\max$ development (Levine and Stray-Gundersen, 1997; Bailey et al., 1998; Gough et al., 2012). Beside potential factors (e.g., iron status, reduction in training qualities despite higher relative intensity) influencing the effects of living and training at altitude, various underlying mechanisms have been suggested (Levine and Stray-Gundersen, 2005). Based on this, it is tempting to associate the improved running velocity with a greater glycolytic metabolism where blood lactate measured after each 400-m effort increased over time and decreased after exercise cessation (2 min of passive recovery). Therefore, contrarily to the physiological adaptations (an increase in $\overset{∙}{V}O_{2}\max$ and no variation in the glycolytic pathway) generally expected at sea level for low aerobic intensity and interval training at v$\overset{∙}{V}O_{2}\max$ (Billat, 2001; MacInnis and Gibala, 2017), the increased running intensity for the same level of $\overset{∙}{V}O_{2}$ induced by the lower oxygen availability at moderate altitude might have participated in an increased anaerobic contribution at the anaerobic threshold and maximal aerobic intensities (Gore et al., 2007; Sharma et al., 2019). The training performed by the present elite female steeplechase runner could have impacted her glycolytic metabolism and improved her capacity to produce more lactate when exercising, as well as a greater buffer capacity during the recovery periods. These specific adaptations observed for the present athlete studied, might be in line with the running intensities sustained during training. Indeed, during altitude training, reduction in absolute altitude training intensity is essential and a meticulous control of training load is key (Mujika et al., 2019). However, some athletes are more affected than others by the lower barometric pressure and oxygen availability at altitude. At 2,100 m altitude, running speed is impaired from 6% to more than 10% for elite athlete (Sharma et al., 2019). Thus, the present athlete studied might have not reduced enough of her running velocity during the altitude camp, inducing more glycolytic adaptations.

From a biomechanical point of view, these results demonstrated that living and training at moderate altitude improved the running velocity and SL. According to Slawinski et al. (2001), within 0–7 m.s^−1^, an increase of the running velocity is mainly associated with an increase in SL. In reference to pre-values, DT_H_ remains constant after intervention. However, the athlete arrives in front of the hurdle with greater VT_H_, resulting in a higher M_H_. To properly clear the hurdle, the athlete must increase M_H_ and DT_H_. Indeed, better hurdlers present a greater take-off distance and a lower take-off angle (Salo et al., 1997). We can therefore speculate that altitude training did not induce any technical adaptations as it was previously demonstrated (Stickford et al., 2017). However, the use of only one athlete without control group clearly limits the interpretation of the present results. Two weeks training period, which offer a potential pathway for further 3,000 m steeplechase performance improvements in highly trained runner. It is more difficult to attribute, with certitude, the observed improvement to the combined effect of training and exposure to hypoxia.

In summary, 2 weeks of training allowed an elite female 3,000-m steeplechase athlete to improve running velocity through a greater glycolytic—but not aerobic—metabolism. However, despite some biomechanical adjustments, specific technical training seems to be necessary in order to improve hurdling technical ability for this specific athlete. These physiological adaptations may be attributed to the benefit of combined training and exposure to hypoxia.

## Data Availability Statement

The datasets generated for this study are available on request to the corresponding author.

## Ethics Statement

Ethical review and approval was not required for the study on human participants in accordance with the local legislation and institutional requirements. The participant provided her written informed consent to participate in this study. Written informed consent was obtained from the individual(s) for the publication of any potentially identifiable images or data included in this article.

## Author Contributions

All authors listed have made a substantial, direct and intellectual contribution to the work, and approved it for publication.

### Conflict of Interest

The authors declare that the research was conducted in the absence of any commercial or financial relationships that could be construed as a potential conflict of interest.
